# Metronidazole Activation by a Deeply Entangled Dimeric Malic Enzyme in *Entamoeba histolytica*

**DOI:** 10.3390/pathogens14030277

**Published:** 2025-03-13

**Authors:** Arindam Chakrabarty, Debajyoti Dutta, Mithu Baidya, Anirudha Dutta, Amit Kumar Das, Sudip K. Ghosh

**Affiliations:** 1 Department of Bioscience and Biotechnology, Indian Institute of Technology Kharagpur, Kharagpur 721302, India; arindamc82@gmail.com; 2 Department of Biotechnology, Thapar Institute of Engineering and Technology, Patiala 147004, India; debajyoti.dutta@thapar.edu; 3 Department of Biosciences and Bioengineering, Indian Institute of Technology Jammu, Jammu and Kashmir 181221, India; mithu.baidya@iitjammu.ac.in; 4 Department of Biological Sciences, Adamas University, Kolkata 700126, India; anirudha1.dutta@adamasuniversity.ac.in

**Keywords:** *Entamoeba histolytica*, metronidazole, crystal structure, entangled protein, knot, protozoa

## Abstract

Metronidazole is the preferred drug for treating amoebiasis caused by *Entamoeba histolytica*. Its antiamoebic activity is primarily attributed to activation by various reductases. This study reports an alternative activation pathway in *E. histolytica* mediated by the decarboxylating malic enzyme. Functional characterization of this NADPH-dependent enzyme reveals that it is secreted into the extracellular milieu and may play a role in *E. histolytica* adhesion to human enteric cells. Structural analysis of the *E. histolytica* malic enzyme (EhME) demonstrates that the protein forms a strict dimer, with the protomers interlocked by a unique knot structure formed by two polypeptide chains. This distinctive structural feature closely aligns EhME with its prokaryotic counterparts. In conclusion, our findings reveal that *E. histolytica* harbors a deeply entangled dimeric malic enzyme that contributes to metronidazole susceptibility, sharing structural similarities with bacterial malic enzymes.

## 1. Introduction

*Entamoeba histolytica* (Eh), the causative agent of amoebiasis, is a leading protozoan parasite responsible for significant mortality in developing countries [1]. Metronidazole (Mtz) remains the most widely used therapeutic agent against amoebiasis. As a prodrug, Mtz is reported to be activated in *Entamoeba* by pyruvate ferredoxin oxidoreductase (PFOR), ferredoxin, thioredoxin reductase, and nitroreductases [2,3]. Interestingly, in *Trichomonas vaginalis* (Tv), a closely related protozoan, studies have shown that even after complete loss of PFOR, cells remain susceptible to higher concentrations of Mtz [4]. However, the development of Mtz-resistant Tv strains was observed after the complete loss of malic enzyme (ME) expression [5]. This finding led to the proposal of an alternative pathway, where a malic enzyme (ME) was proposed to provide electrons for Mtz activation in the absence of PFOR. In this alternative pathway, the oxidative decarboxylation of malate to pyruvate via the hydrogenosomal malic enzyme, coupled with NADH-ferredoxin oxidoreductase (which reoxidizes NADH to NAD⁺), transfers reducing equivalents to ferredoxin. Ferredoxin, in turn, reduces the nitro group of Mtz, producing Mtz radicals [4]. These cytotoxic radicals bind to DNA [6] and protozoan proteins [2], causing DNA breakage and protein adducts that ultimately lead to cell death [7].

Malic enzyme (ME) catalyzes the conversion of malate to pyruvate through a two-step reaction. In the first step, malate is oxidized to oxaloacetate, which is subsequently decarboxylated to pyruvate and CO_2_ [8]. The sequence, structure, and oligomerization of malic enzyme (ME) exhibit significant variability across living systems [9,10]. Three isoforms of ME are commonly found. The NADP⁺-dependent malic enzyme (NADP-ME; EC 1.1.1.40) exhibits a preference for NADP⁺ over NAD⁺ and has the unique ability to decarboxylate oxaloacetate to pyruvate directly [11,12]. The NAD⁺-dependent malic enzyme (NAD-ME; EC 1.1.1.39) is specialized for decarboxylating malate to pyruvate [13]. A third isoform, another NAD⁺-dependent enzyme (EC 1.1.1.38), can decarboxylate oxaloacetate and utilize both NADP⁺ and NAD⁺, though it preferentially uses NAD⁺ [14]. ME can also be categorized based on subcellular localization or oligomerization. For instance, plant NAD⁺-dependent mitochondrial ME is phylogenetically distinct from the NADP⁺-dependent cytosolic and plastidial forms found in plants [10]. The functional roles of ME vary widely across organisms. In humans, ME plays a critical role in providing energy to neurons and supporting proliferating cells [15,16]. In *E. histolytica* (Eh), this pathway plays a role in fermentation, contributing to energy generation [17], as in many other eukaryotes. A close homolog of *E. histolytica* malic enzyme (EhME) is AP65 (TvAP65), a hydrogenosomal NADP^+^-dependent ME found in *T. vaginalis* (Tv). Beyond its housekeeping role in pyruvate metabolism, TvAP65 has developed an additional function as an adhesive protein. It is secreted into the extracellular environment, where it facilitates *T. vaginalis* adhesion to host cells [18,19]. Whether EhME performs a similar function remains an open question. In this study, we have biochemically and structurally characterized the NADP⁺-dependent EhME, identifying its role in metronidazole (Mtz) activation. Furthermore, we have uncovered evidence suggesting that EhME may play a potential role in host–cell interactions. A structural analysis of EhME reveals a unique physical knot distinct among eukaryotic MEs. The evolution of this structural feature may support its involvement in cell adhesion, potentially contributing to the initiation of *E. histolytica* pathogenesis.

## 2. Materials and Methods

### 2.1. Cloning, Over-Expression, and Purification

The *E. histolytica* malic enzyme gene (GenBank accession no. AF234166) was PCR-amplified from genomic DNA using gene-specific primers: forward primer 5′-GGA TCC ATG GCA CAA TTA AAA GCA GAT CT-3′ and reverse primer 5′-CTC GAG TCA TTT TCC AGT GAC TTT GTT AAT AG-3′. The resulting amplicon was initially cloned into the pTZ57R/T vector (Fermentas, Waltham, MA, USA) and subsequently recloned into the pQE30 expression vector (Qiagen, Hilden, Germany), which contained an N-terminal hexahistidine tag. This recloning process utilized *Bam*HI and *Sal*I restriction sites incorporated into the forward and reverse primers, respectively. The recombinant plasmid was expressed in *E. coli* strain M15. For protein purification, the cells were resuspended in a lysis buffer (RB) containing 20 mM of Tris-Cl, 300 mM of NaCl, and 20 mM of imidazole at a pH of 8.0. The cells were lysed by sonication, and the lysate was centrifuged at 14,000 rpm for 30 min. The resulting supernatant was loaded onto a pre-packed Ni-NTA column prewashed with the same RB. The column was then washed with five column volumes of RB supplemented with 50 mM of imidazole, and the bound protein was eluted using RB containing 150 mM of imidazole. The eluted protein was further purified by Superdex 200 gel exclusion chromatography, which had been pre-equilibrated with a buffer containing 10 mM of Tris, 150 mM of NaCl, and 10 mM of dithiothreitol (DTT) at a pH of 8.0. The purity of the protein was assessed using SDS–PAGE, and the final purified protein was concentrated to 50 mg/mL using an Amicon concentrator (Millipore, Burlington, MA, USA). The concentration was estimated using the Bradford method, and absorbance was measured at 280 nm (A_280_).

### 2.2. Biochemical Characterization

The ME activity was performed using recombinant EhME involving the NADP+ dependent oxidation of L-malate. The assay was performed in a Varian Cary50 UV/Vis spectrophotometer using a quartz cuvette with a 1 cm path length (Varian Medical Systems, Palo Alto, CA, USA). The standard reaction mixture consisted of 50 mM Tris-Cl (pH 7.4), 5 mM MnCl_2_, 10 mM L-malate, 0.3 mM NADP⁺, and 1 μg of protein in a final volume of 750 μL. After the enzyme was added to the reaction mixture at 30 °C, the increase in absorbance was continuously monitored at a wavelength of 340 nm. A molar extinction coefficient of 6.22 mM^−1^ cm^−1^ for NADPH was used to calculate enzyme activity. One unit of enzyme activity was defined as the amount of enzyme required to catalyze the production of 1 μmol of NADPH per minute. The apparent Michaelis-Menten constants (Km) for the substrates L-malate and NADP⁺ were determined by varying the concentration of either L-malate (5–15 mM) or NADP^+^ (0.1–0.5 mM) while keeping other components at their saturation levels. To determine the binding mechanism of NADP^+^ and L-malate, Lineweaver-Burk (LB) plots were generated at different concentrations of NADP^+^ (0.2, 0.5, and 0.8 mM) with variable concentrations of L-malate (5–15 mM). The ATP inhibition of the malic enzyme was also evaluated using varying concentrations of ATP (0.1–1.0 mM) in the presence of different concentrations of L-malate (5–20 mM) and NADP^+^ (0.1–1.0 mM). All kinetic assays were performed at least three times and in triplicate, and the reported values represent the averages of these replicates. Finally, the inhibition constants (K_i_) were determined. All results were analyzed using OriginLab software, (Origin 8.5).

### 2.3. Metronidazole Susceptibility Assay

The involvement of EhME in metronidazole activation was assessed using two different methods: broth dilution assay and E-test.

### 2.4. A. Broth Dilution

Overnight cultures of metronidazole-sensitive *E. coli* strain JM109 containing the plasmid pQE30, with and without EhME inserts, were inoculated and subsequently induced with 0.5 mM IPTG for 5 h. Approximately 50 μL of the induced cultures were transferred into prewarmed 3 mL LB media supplemented with 100 μg mL^−1^ ampicillin and grown at 37 °C for 1 h. The cultures were then exposed to varying concentrations of metronidazole, ranging from 0 to 320 μg mL^−1^, and incubated for an additional 12 h. The optical density (OD) for bacterial growth in the overnight-grown culture was measured using a UV-visible spectrophotometer (DU 500, Beckman, Brea, CA, USA) at 600 nm. *E. coli* without any EhME expression was used as a control. At least three independent experiments were performed in triplicate. A graph was plotted, and the minimum inhibitory concentration (MIC) was calculated using GraphPad Prism software (Version 5.0).

### 2.5. B. E-Test Assay

The EhME-induced bacterial inoculum was prepared in the same manner as the broth dilution assay. Six milliliters of sterilized top agar at 42 °C, supplemented with 100 μg mL^−1^ ampicillin, was mixed with 200 μL of log-phase grown EhME-induced bacteria and overlaid on LB agar plates. *E. coli* without any EhME expression was used as a control. After the plates had dried, Mtz E-strips were aseptically placed at the center of the plates. The plates were incubated at 37 °C for 12 to 16 h in an upright position. The minimum inhibitory concentration (MIC) was determined from the intersection point of the inhibitory zone and the E-strip. Each set of experiments was conducted for three independent experiments and in at least triplicate.

### 2.6. Cell Culture and Drug Treatment

The human colon cancer cell lines [Caco-2] obtained from ATCC (ATCC HTB-37) and HT29 (ATCC HTB-38) were cultured in Dulbecco’s modified Eagle’s medium (HiMedia, India) and RPMI (HiMedia, Mumbai, India), respectively, supplemented with 10% FBS (HyClone, Logan, UT, USA) and 1% antibiotics (Ampicillin and Streptomycin, HiMedia, Mumbai, India) at 37 °C in a 5% CO_2_ incubator. *E. histolytica* trophozoites (HM1:IMSS isolate) were cultured axenically in TYI-S-33 medium supplemented with 10% heat-inactivated bovine serum. Metronidazole was added to a final concentration of 5 μM and 12 μM to mid-logarithmic phase trophozoites, and trophozoites without drug treatment were used as controls.

### 2.7. Semi-Quantitative RT-PCR

For transcriptional analysis, *E. histolytica* cells (146 strain) were treated with 5 and 12 μM metronidazole for 6 h, and untreated cells were used as controls. Total RNA was isolated from metronidazole-treated and untreated cells using TRI reagent (Ambion, Austin, TX, USA) according to the manufacturer’s protocol. First-strand cDNA was synthesized from isolated RNA using the RETROscript kit (Ambion, Austin, TX, USA), following the manufacturer’s instructions. The synthesized cDNAs were amplified by PCR using Taq polymerase (NEB, Ipswich, MA, USA), with the PCR conditions and primers the same as those used for EhME amplification from genomic DNA. The PCR amplification was verified by quantifying the cDNA-PCR products obtained after 25 cycles, ensuring amplification was within the linear range of the corresponding mRNA species. The products were analyzed using agarose gel electrophoresis, and densitometric quantification was performed using UVP Launch Doc ITLS software (UVP, Jena, Germany). All amplified RT-PCR products were normalized with respect to actin RT-PCR products.

### 2.8. Western Blot Assay

Proteins were separated on a 12% SDS-PAGE gel, which was then transferred to a nitrocellulose membrane. The membrane was probed with an anti-His monoclonal antibody (1:2000; Sigma, Cream Ridge, MA, USA) and an in-house generated antibody against EhME (1:100), followed by HRP-conjugated anti-mouse (1:5000) and anti-rabbit secondary antibodies (1:5000; Sigma, Cream Ridge, MA, USA). Detection was carried out using ECL (Millipore, Burlington, MA, USA). Actin was used as a loading control.

### 2.9. Labeling of Recombinant Malic Enzyme

Purified EhME (2 mg mL^−1^) was incubated with 1 mg mL^−1^ FITC in 0.5 M carbonate buffer (pH 9.5) at room temperature for 1 h with gentle mixing. The mixture was dialyzed overnight against 1X PBS at 4 °C, with three buffer changes. The labeled protein was then stored at −20 °C for future use.

### 2.10. Preparation of Cell-Free Filtrate

Exponentially growing Eh cells were harvested and resuspended to a density of 5 × 10^6^ cells mL^−1^ in PBS + 10 mM glucose (PBS-M) at pH 5.5, 6.0, and 6.8 and incubated at 37 °C for 2 h to allow secretion. Trypan blue exclusion assays were performed at the end of incubation to check cell viability, and batches with >99% viability were selected for further study. Supernatants from these batches were collected and filtered through a 0.45 μm syringe filter. The cell-free supernatant was concentrated using a 10 kDa cut-off Centricon unit (Millipore, USA), and the concentrated protein was analyzed by Western blotting.

### 2.11. Detection of Extracellular Released EhME Protein

Trophozoites (10^7^ cells mL^−1^) at the mid-log phase of growth were washed in TYI-S-33 medium, resuspended in the same medium, and incubated for an additional 12 or 18 h at 37 °C. Parasite viability was monitored using a trypan blue exclusion assay, and cell lysis was confirmed by microscopic observation. Supernatants were collected by centrifugation at 1400 rpm at 4 °C. The resulting supernatant was filtered through a 0.22 μm filter to remove insoluble debris, then precipitated with 10% TCA (*w*/*v*) and incubated overnight at 4 °C. The precipitate was centrifuged for 10 min at 10,000 rpm, washed twice with 4 mL acetone, and dried in air. The dried proteins were resuspended in 1X PBS and analyzed by Western blot.

### 2.12. Localization of Malic Enzyme in Eh

Cells were washed three times with 1X PBS and fixed with 2% paraformaldehyde for 10 min on ice. After fixation, the cells were washed again three times with 1X PBS and permeabilized with 0.01% Triton X-100. The cells were then incubated with a specific anti-EhME antibody raised in rabbit (1:100 dilution) for 1 h at room temperature, followed by incubation with an anti-rabbit IgG TRITC-conjugated secondary antibody (1:500). The cells were subsequently stained with DAPI (1 μg m^−1^) for nuclear staining, washed with 1X PBS, and visualized by confocal microscopy (FV 1000, Olympus, Japan). Images obtained were analyzed and processed using FluoView1000 software.

### 2.13. Autoligand Assay

An auto-ligand assay was performed to test the binding ability of recombinant EhME with its own membrane. Approximately 2 × 10^4^ Eh cells per well were grown in a 24-well plate for 4 h. One set of cells was treated with trypsin (10 mg mL^−1^) for 15 min at 37 °C, while the other was untreated. The cells were then fixed with 2% paraformaldehyde for 10 min. The FITC-labeled recombinant enzyme was added to the cell monolayer and incubated at 37 °C for 1 h. In another experiment, cells were grown at pH 6.0 for 1 h and then stained with anti-EhME antibody, followed by TRITC-labeled secondary anti-IgG rabbit antibody as described above. The samples were visualized under a confocal microscope.

### 2.14. Adherence Assay

For the adherence assay, human colon cancer cell lines HT-29 and Caco-2 were grown as monolayers. Caco-2 cells were cultured for 4 days, while HT-29 cells were cultured for 12–18 h. Prior to adding the labeled protein, the cells were washed with 1X PBS, and 20 μg mL^−1^ labeled protein was added to each well and allowed to bind for 1 h at room temperature. In one set of experiments, HT-29 cells were incubated with spent media separated from Eh cell cultures grown at pH 6.0 for 2 h. Non-specifically bound EhME was washed away with 1X PBS, and the cells were fixed with 2% paraformaldehyde. The cells were then observed under a confocal microscope.

### 2.15. Crystallization

Concentrated EhME (50 mg mL^−1^) was subjected to preliminary crystallization trials with Crystal Screen and Crystal Screen 2 (Hampton Research, Aliso Viejo, CA, USA), where 2 μL of protein was mixed with 2 μL of reservoir solution in the sitting drop vapor diffusion method at 298 K. Tiny crystals appeared in 2.0 M (NH_4_)_2_SO_4_. An attempt was made to improve the quality of the crystals by introducing acetate buffer. Fine screening with pH ranges from 5.0 to 5.5 and varied concentrations of (NH_4_)_2_SO_4_ in the hanging drop vapor diffusion method were carried out. Good quality crystals appeared from the condition consisting of 1.5 M (NH_4_)_2_SO_4_ and 0.1 M CH_3_COONa at pH 5.0 after 7 days at 298 K. A platinum heavy atom derivative of the crystal was prepared by soaking in 2 mM Na[Pt(CN)_4_].

### 2.16. Data Collection and Processing

EhME crystals were quickly soaked in a crystal solution containing 1.5 M (NH_4_)_2_SO_4_, 0.1 M CH_3_COONa at pH 5.0, and 12% (*v*/*v*) glycerol and mounted. X-ray diffraction data were collected on an in-house Rigaku RaxisIV^++^ image plate detector using CuKα X-rays generated by the Rigaku Micromax HF007 rotating anode generator in a nitrogen stream at 100K (Rigaku, Tokyo, Japan). The crystal-to-detector distance was kept at 180 mm and rotated a total of 360°, with 0.5° oscillation per frame. The native crystal diffracted up to a resolution of 2.2 Å. A total of 720 frames were collected and processed with d*TREK [20] with space group P4_3_2_1_2. Heavy atom data were collected using the same in-house diffractometer. Data processing statistics are summarized in Table 1.

### 2.17. Phasing, Model Building, Refinement, and Validation

Attempts to obtain the phase from homologous structures using molecular replacement failed. The phases were obtained using the SIRAS method using the platinum derivative. The heavy atom substructure was determined by SHELXD [21]. AutoSHARP automatically interpreted initial sites and calculated residual maps [22]. Density modification was performed using SOLOMON [23], followed by automatic model building using ARP/wARP. The resulting model, which contained 80% of the total residues in the asymmetric unit, was further subjected to manual model building in COOT [24] and refined to R_work_ and R_free_ values of 17.9% and 24.4%, respectively, in REFMAC [25]. Detailed phasing and refinement statistics are summarized in Table 1. The final model was validated using PROCHECK [26] and SFCHECK in the CCP4. The atomic coordinates and experimental structural factor amplitudes for EhME have been deposited in the Protein Data Bank (PDB), with accession number 3NV9.

### 2.18. Computational Docking Analysis

Computational docking of ATP to EhME was performed independently using Autodock [27] and the Swissdock server [28]. Ligand (ATP) and receptor (EhME; PDB ID 3NV9) coordinates were retrieved from the PDB and used for blind docking after energy minimization. To employ the EhME dimer in the docking process, the entire EhME dimer was selected as the receptor. Docking clusters were selected based on the lowest free energy of the complex.

## 3. Results

### 3.1. Malic Enzyme Is an NADP^+^ Dependent Enzyme

**Figure 1:**
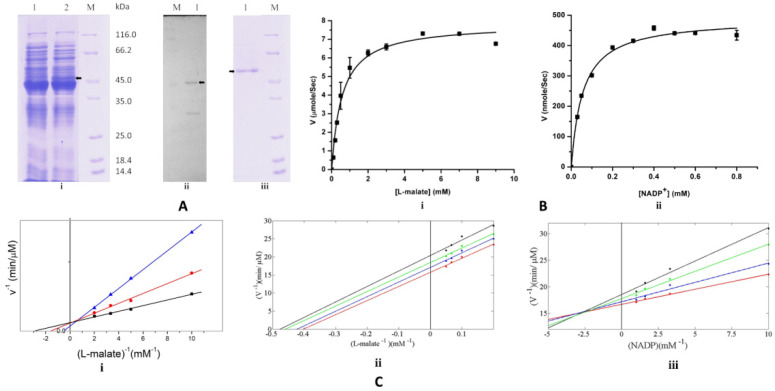
Biochemical characterization of EhME. (**A**). Protein expression and purification on 12% SDS-PAGE; (i) lane 1 contains uninduced *E. coli* M15 lysate and lane 2 contains the induced, (ii) Western blot of EhME with anti-His antibody, (iii) protein purified to homogeneity by gel filtration, M represents the protein marker. Black arrows indicate the expressed EhME. (**B**). Kinetic analysis of EhME (i) in different concentrations of L-malate. (ii) in different concentrations of NADP^+^. (**C**). EhME interaction with NADP^+^, L-malate, and ATP (i) EhME interaction with NADP^+^ and L-malate is bi-bi interaction, inhibition of EhME by ATP. NADP^+^ concentrations of 0.2 mM (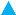), 0.5 mM (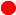), and 0.8 mM (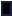) are kept fixed to determine the reaction velocities with respect to the different L-malate concentrations, (ii) uncompetitive inhibition of the EhME by ATP with respect to malate, (iii) non-competitive inhibition of the EhME by ATP with respect to NADP^+^. Malic enzyme activity was measured at different concentrations of NADP^+^ in various concentrations of ATP (indicated by different color). The X-axis indicates the concentration of the substrate, and the Y-axis indicates the velocity of the reaction.

For biochemical characterization, EhME was heterologously overexpressed in bacterial cells. The purified EhME exhibited an apparent molecular mass of 53 kDa on 12% SDS–PAGE, and its identity was further confirmed through Western blot analysis using an anti-His antibody (Figure 1A). The purified enzyme catalyzed the oxidative decarboxylation of L-malate to pyruvate, utilizing NADP⁺ as a coenzyme. The Michaelis-Menten constants (Kₘ) for L-malate and NADP⁺ were determined to be 0.57 ± 0.08 mM and 46.67 ± 6.0 μM, respectively. The catalytic turnover rates (k_cat_) were 41.7 s⁻¹ for L-malate and 14.57 s⁻¹ for NADP⁺ (Figure 1B). The k_cat_ values are determined by dividing maximum reaction velocity (V_m_) with the total enzyme concentration [E_t_] where the V_m_ for the L-malate and NADP⁺ were determined to be 7.84 ± 0.26 mM s^−1^ and 2.75 ± 0.12 mM s^−1^, respectively. Lineweaver-Burk plots generated at varying concentrations of L-malate and fixed NADP⁺ concentrations (0.2, 0.5, and 0.8 mM) showed changes in both slope and intercept (Figure 1C), indicating that EhME catalyzes the oxidative decarboxylation of L-malate via a sequential bi-bi (ternary complex) reaction mechanism. These findings confirm that the recombinant EhME was purified in an active form. The kinetic parameters are within similar ranges of the other malic enzymes studied [29,30]. Since ATP is known to inhibit malic enzymes (e.g., human ME), ATP-mediated inhibition of EhMEwas assessed. The inhibition pattern of EhME by ATP was non-competitive with respect to NADP⁺ and uncompetitive with respect to L-malate. The inhibition dissociation constants (Kᵢ) for ATP were 1.63 mM for NADP⁺ and 0.65 mM for L-malate (Figure 1C). These results suggest that ATP binds to an allosteric site distinct from the NADP⁺ binding site, with the inhibitory effect being influenced by L-malate binding to EhME.

### 3.2. Malic Enzyme Is an Alternative Enzyme That Activates Metronidazole

**Figure 2:**
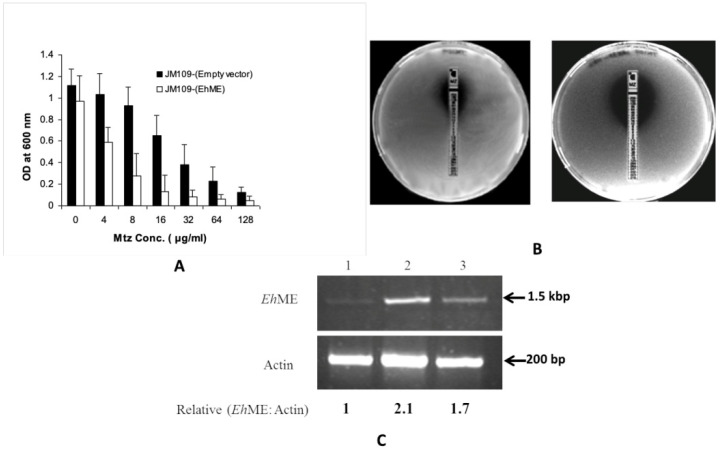
Metronidazole activation by EhME and its effect on *E. histolytica*: (**A**) Overexpression of EhME in *E. coli* (JM109 strain) increases the metronidazole sensitivity. Response to different concentrations of metronidazole to transformed *E. coli* strain JM109 with empty vector (pQE30) and pQE30-EhME was measured by broth dilution method. (**B**) E-test for metronidazole sensitivity (i) JM109+ empty vector (ii) JM109+ pQE30-EhME. (**C**) Semi-quantitative RT-PCR analysis of malic enzyme expression. RT-PCR was performed from total RNA isolated form untreated (1), metronidazole treated 5 µM (2) and 12 µM (3) cells. The RT-PCR products were verified on 1.5% agarose gel. Black arrows indicate the corresponding base pair positions.

Various oxidoreductases have traditionally been considered the primary enzymes responsible for activating metronidazole (Mtz). However, their loss did not render *E. histolytica* (Eh) or *T. vaginalis* (Tv) cells resistant to higher doses of Mtz, suggesting the existence of an alternative pathway potentially involving malic enzyme (ME). The ability of EhME to reduce Mtz was tested using two complementary methods: (i) broth dilution assay and (ii) E-test. An Mtz-sensitive *E. coli* JM109 strain was transformed to ectopically express EhME. The JM109 cells transformed with the empty pQE30 vector showed an IC_50_ value of 32 μg mL^−1^. In contrast, cells expressing EhME displayed a significantly reduced IC_50_ of 6.25 μg mL^−1^ (Figure 2A). These results were further validated using the Epsilometer test (E-test; AB Biodisc, Sweden). When an Mtz E-strip containing a gradient of drug concentrations (0.156–256 μg mL^−1^) was applied, a distinct zone of inhibition was observed in plates with JM109 cells expressing EhME, whereas the control plates with cells transformed with the empty vector showed no comparable effect (Figure 2B). To investigate whether sustained Mtz activation in Eh is linked to the drug influencing EhME expression, semi-quantitative RT-PCR (sqRT-PCR) was performed on cells treated with 5.0 mM and 12 mM of Mtz. The analysis revealed that treatment with 5.0 mM Mtz increased EhME expression by over 2.1-fold compared to untreated cells.

In cells treated with 12.0 mM Mtz, EhME expression increased by 1.7-fold, likely due to cell death caused by the higher drug concentration (Figure 2C). A similar trend was observed in Western blot analysis (Figure S1). These findings suggest that Mtz activation in Eh is a self-driven process where Mtz itself induces the expression of EhME, enabling its metabolism. The reduction in metronidazole in the presence of malate, NADH dehydrogenase, and ferredoxin could be confirmed by EPR analysis.

### 3.3. Malic Enzyme Helps Attach Eh to the Enteric Cell Surface

**Figure 3:**
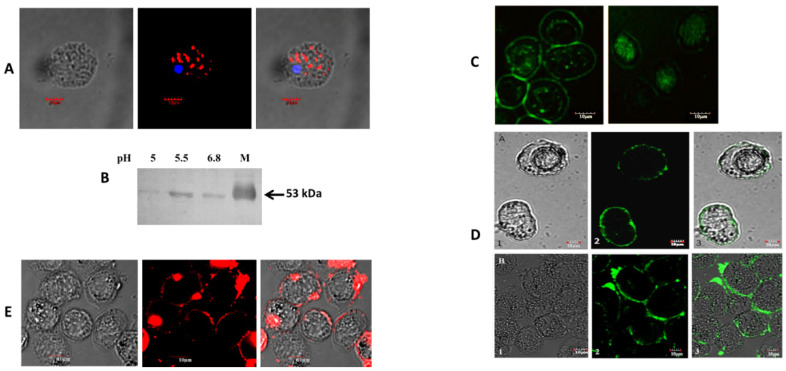
EhME assists in the attachment of Eh to human enteric cells. (**A**). localization of the malic enzyme in Eh cells: Overnight grown cells were fixed, permeabilized, and incubated with anti-EME antibody and TRITC conjugated anti-rabbit IgG secondary antibody. The red color puncta represents vesicles containing EhME, and the blue color (DAPI) represents the nucleus of the cell. (**B**). The Western blot of extracellular malic enzyme, released by cells at different pH. Lane M, purified recombinant EhMalik enzyme as molecular weight reference. (**C**). Autoligand assay: Eh cells were incubated with FITC labeled recombinant EhME for identifying any surface receptor if it possesses specific to EhME, (i) normal Eh cells; (ii) trypsin treated Eh cells. (**D**). Binding of FITC labeled recombinant EhME to human colon cell line. Human colon cell HT29 and Caco-2 cells were stained with FITC-labeled recombinant EhME. (**E**). Binding of extracellular released malic enzyme with HT29 cells. Cells were incubated with extracellular EhME released at pH 6.0 and then stained for EhME.

In recent studies, a hydrogenosomal NADP⁺-dependent decarboxylating malic enzyme from the protozoan *T. vaginalis*, known as AP65, has been implicated in adhesion to host cells [19]. A pairwise alignment revealed that TvAP65 exhibits significant sequence similarity to EhME (Figure S2), which is the single malic enzyme isoform encoded by the Eh genome. To determine if EhME plays a similar role, we investigated its intracellular distribution through immunolocalization. Confocal microscopy revealed that EhME is predominantly localized in the cytosol as distinct puncta (Figure 3A). Many adhesion-associated proteins are secreted in response to environmental triggers, such as a decrease in pH [31]. The release of EhME into the extracellular environment was assessed via Western blot analysis at different ambient pH levels, with maximum secretion detected at pH 6.0 (Figure 3B).

To explore whether the released EhME could attach to a host and Eh cells, we performed an autoligand assay in which non-permeabilized Eh cells were incubated with FITC-tagged recombinant EhME (rEhME). Confocal microscopy confirmed that rEhME firmly bound to the surface of Eh cells but failed to bind to the surface of trypsin-treated cells (Figure 3C). Additionally, similar experiments were conducted on human enteric colon cells, including HT29 and Caco-2 cells. Immunolocalization demonstrated that rEhME was firmly bound to the surface of these colon cells (Figure 3D). In contrast, no binding was observed on MCF cells, which served as a negative control. To confirm whether low pH-induced secretion of EhME by Eh cells could bind human enteric cell surfaces, Eh cells were grown at pH 6.0, and the spent media were collected and incubated with HT29 cells for 1 h. Immunolocalization showed that the secreted EhME successfully attached to the surface of HT29 cells (Figure 3E). These findings suggest that EhME plays a dual role in maintaining the attachment by binding to both host and pathogen cells, highlighting its potential role as a key participant in adhesion processes.

### 3.4. Overall Structure of EhME

**Figure 4:**
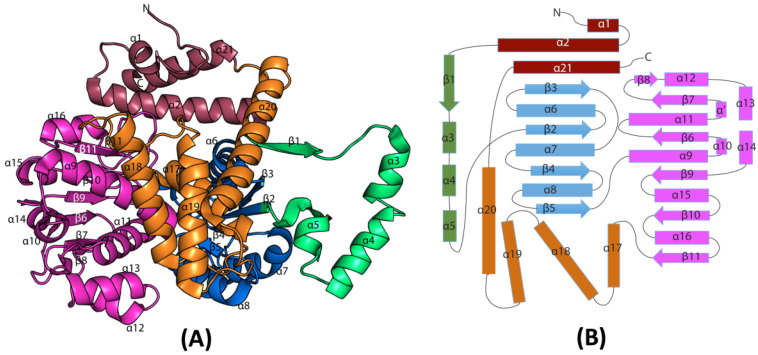
The overall structure of EhME. (**A**). Cartoon representation of EhME monomer. (**B**). The topology diagram of EhME. Based on the domain region, the secondary structures are put together and colored.

The crystal structure of EhME was determined at a resolution of 2.2 Å using the platinum single isomorphous replacement with anomalous scattering (SIRAS) method. The crystal belongs to space group P4_3_2_1_2, with two nearly identical monomers in the asymmetric unit, exhibiting an RMSD of 0.35 Å across 3581 atoms (Figure 4A). The dimeric nature of EhME was further validated through gel filtration chromatography. The monomer structure is organized into five distinct domains (Figure 4B). The shaft-like domain A comprises two N-terminal helices, α1 (residues 10–15) and α2 (residues 20–44), along with one C-terminal helix, α21 (residues 470–485). Dimer interface domain B includes helices α3 (residues 58–64), α4 (residues 67–78), and α5 (residues 82–90). These helices intertwine with the second monomer, forming a knot that stabilizes the active site through inter-monomer β1-β1′ interactions. Substrate-binding domain C spans residues 92–193 and features four parallel β-strands (β5, β4, β2, and β3) flanked by helices α6–α8. The NADPH binding domain D (residues 194–359) exhibits a Rossmann fold typical of coenzyme-binding regions. It contains five central parallel β-strands (β11, β10, β9, β6, and β7) surrounded by helices α9–α16. Finally, the helix bundle domain E, an all-helical domain (residues 360–469), consists of helices α17–α20, forming a three-helix bundle. The extended α20 helix (residues 438–467) aligns with the dimer interface and connects to α21.

### 3.5. The Entangled Dimer Interface of EhME Is Distinctive from the Metazoan ME

**Figure 5:**
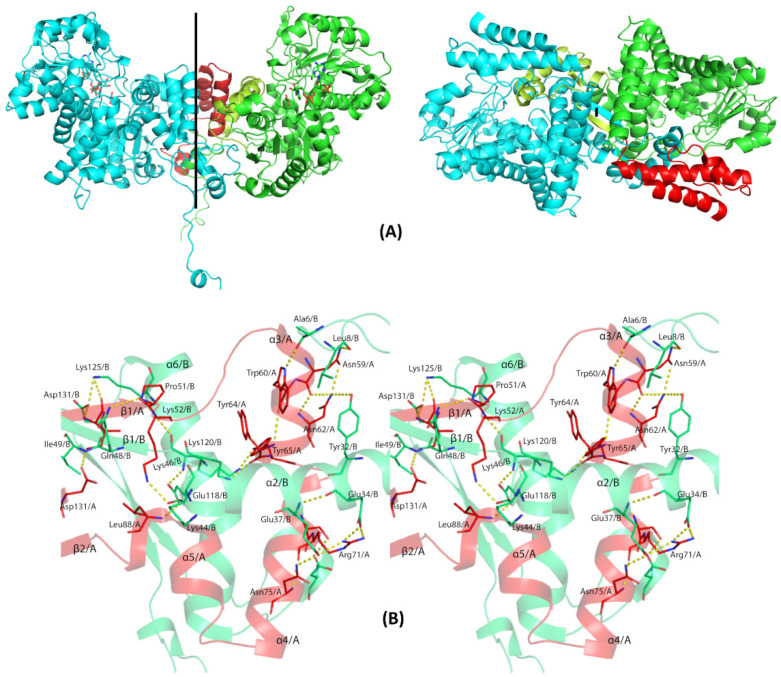
Quaternary structure of EhME. (**A**). Difference between the dimer organizations of eukaryotic ME (left) and EhME (right). The black line indicates the axis of two-fold symmetry. Two monomers are indicated by green and magenta color. Domain A shaft and domain B of green monomer are colored red and lime green, respectively, to indicate the difference between the spanning helices through intra- (in metazoan) and inter- (in EhME) monomer. (**B**). Wall-eyed stereo view of the knot region of the dimer. Different monomers (**A**) and (**B**) are indicated by different colors. The inter-monomer spanning region starts with β1/A-β1/B interaction, and spanning helices α3-α4-α5 pass through the domain A shaft and the core domain C.

**Figure 6:**
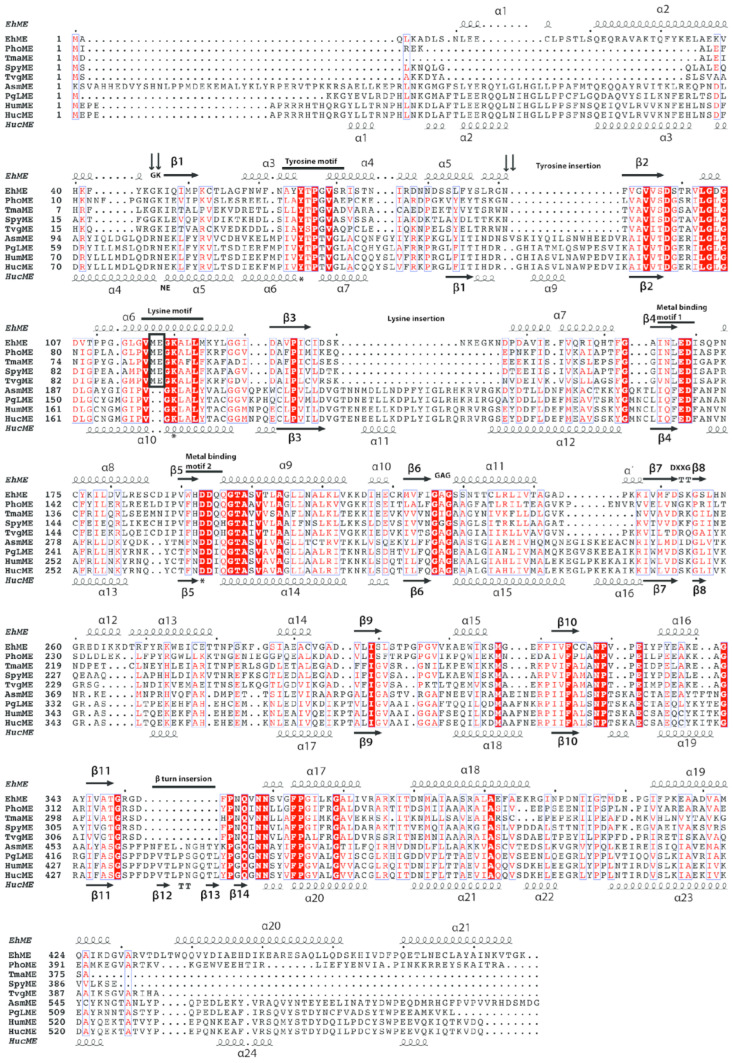
Structure sequence alignment of EhME. EhME: EhME: *Entamoeba histolytica* malic enzyme (Q9NH04); PhoME: *Pyrococcushorikoshii* malic enzyme (O59029); TmaME: *Thermotoga maritima* malic enzyme (Q9WZ12); SpyME: *Streptococcus pyogenes* malic enzyme (Q99ZS1); TvgME: *Trichomonas vaginalis* malic enzyme (A2F6B9); AsmME: Ascaris suum malic enzyme (P27443); PgLME: Pigeon liver malic enzyme (P40927); HumME: Human mitochondrial malic enzyme (P23368); HucME: Human Cytosolic malic enzyme (P48163). Secondary structures of EhME (3VN9) and HucME (3WJA) are presented at the top and the bottom of the sequence alignment, respectively. The active sites Tyr, Lys, and Asp are highlighted using asterisks. A Knotted portion of the bacterial malic enzymes are marked by down arrows (^45^GK^46^ and ^90^GNF^92^). The tyrosine and lysine insertions in eukaryotic MEs are indicated. The intermonomer spanning helices α3-α4-α5 in prokaryotic/EhME are changed into intramonomer α5-α6-α7 in metazoan MEs. Alteration near the lysine motif is indicated by the black box. Metal binding motifs, GAG, and DXXG motifs harboring sulfate moiety are also indicated. The β turn insertion (β12-β13) insertion in eukaryotic ME is in proximity to the nicotinamide region. The red letters refer to the amino acids with similar properties, and the white letters with red background refer to the conserved amino acids.

Unlike the tetrameric configuration observed in many eukaryotic malic enzymes, EhME exists strictly as a dimer, as confirmed by gel filtration chromatography (Figure 5A). Prokaryotic malic enzymes with sequence identities of 43–47% to EhME also form dimers or tetramers; however, the dimeric forms uniquely exhibit an inter-protomer entangled polypeptide at their interface. This entangled conformation appears to be an inherent structural feature of prokaryotic MEs, including EhME and is independent of crystallization conditions or space groups (Figure 5B and Figure S3). The dimer is stabilized by a two-fold symmetry axis perpendicular to the inter-dimer β1-β1′ sheet. The entangled region begins at the EhME-specific motif ^46^GK^46^, where the polypeptide chain of one monomer enters the other protomer, forming the β1-β1′ interaction. Helices α3, α4, and α5 from one monomer pass through the domain A and the core structure of the other monomer before re-entering their respective structures near the N-terminus of β2 (sequence ^90^GNF^92^). This creates a 48-residue entangled region. The axes of helices α3, α4, and α5 are oriented perpendicularly to their counterparts in the opposing monomer (α3′, α4′, and α5′). Sequence alignments between eukaryotic and prokaryotic MEs reveal significant structural differences in the region spanning from the N-terminus of β1 to the C-terminus of α7 in EhME (Figure 6). Notably, the ^45^GK^46^ motif in EhME is replaced by NEK in eukaryotic MEs.

Additionally, eukaryotic MEs contain two insertions—termed the “tyrosine insertion” and “lysine-insertion”—absent in EhME. These insertions likely evolved to avoid the formation of the entanglement in eukaryotic enzymes. In EhME, the absence of these insertions results in altered interaction patterns, converting intra-monomer interactions seen in eukaryotic MEs into inter-monomer interactions. For example, the inter-monomer spanning helices (α3, α4, and α5) of one protomer interact with the ^44^KGKI^47^ region of the opposing protomer (Figure 5B). The swapping of secondary structure positions across the termini of α3–α4–α5 facilitates the β1-β1′ interaction. Detailed residues involved in the knot region are summarized in Table 2.

### 3.6. The Composite Active Site of EhME

**Figure 7:**
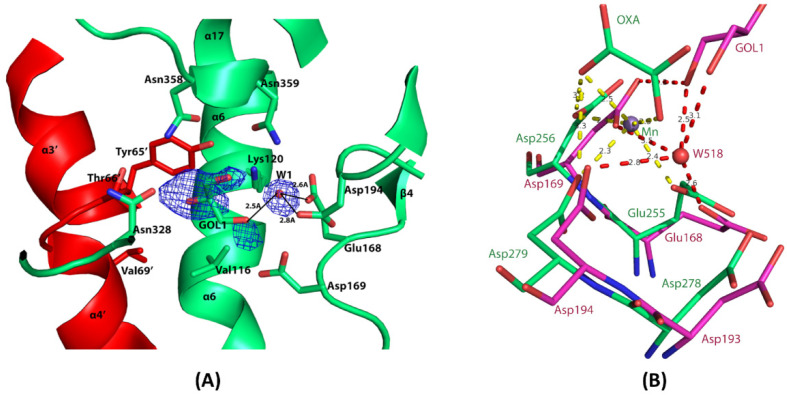
The composite active site and metal binding site of EhME. (**A**). Monomers are designated by different colors. Catalytic Tyr^65′^ is located on the intermonomer spanning α3′–α4′ region. Catalytic Lys^120^ is situated on α6, and Asp^193^ is a part of the metal binding motif 2 (β5–α9 loop). A glycerol molecule, GOL1, at the overlapping position of the substrate binding region, is fitted in the Fo-Fc difference density contoured at 2.8 σ cut-offs. (**B**). EhME (green) metal binding site is compared with PglME (magenta). The substrate mimics OXA (oxaloacetate) and occupies the PglME active site. The Mn^2+^ of PglME is shown.

The catalytic triad of EhME is composed of Tyr^65^, Lys^120^, and Asp^193^. Tyr^65^ and Lys^120^ are part of the Tyr and Lys motifs, respectively. Due to the knotted structure of EhME, two monomers engage in inter-monomer swapping of these motifs, contributing to the formation of each other’s active site (Figure 5B). The Tyr motif (^65^YTPGV^69^), located between α3′ and α4′, is positioned near Lys^120^ as a result of the knotted structure (Figure 7). In contrast, metazoan malic enzymes (MEs) lack this inter-monomer knot and each monomer possesses its own distinct active site. The Tyr motif in metazoan MEs is typically “YTPTV”, with a key difference at the fifth position where Gly is replaced by Thr. This alteration may be essential for ensuring the correct orientation of helices α6 and α7 in metazoan MEs. The EhME Lys motif (^116^VMEGKA^121^), located within helix α6, includes Glu^118^, which forms hydrogen bonds with the ^45^GK^46^ motif and the Cα backbone of α5. A comparison of the structures of metazoan MEs with EhME reveals that the inter-monomer spanning region (α3, α4, and α5) in EhME is replaced by the α5–α6–α7 intra-monomer region in metazoan MEs.

This structural divergence suggests that the shortening of the N-terminal sequence in EhME might be responsible for its more rigid, compact structure and proper orientation of the catalytic residues, which is essential for knot formation. The reason for maintaining a composite active site in EhME remains unclear, but one possibility is that it serves to preserve structural compactness. Confocal imaging indicates that the enzyme is secreted into the intercellular milieu, an environment subject to mechanical stress and fluctuating conditions. Unlike metazoan MEs, EhME does not have long loop insertions and has more structured N- and C-terminal regions. In fact, these regions in EhME are even more structured and elongated compared to the N- and C-terminal regions observed in prokaryotic MEs (as seen in PDB structures). The structured nature of these regions cannot be attributed to crystal contacts, further suggesting that they play an important role in the enzyme’s stability. The knotted-dimer structure further strengthens this compactness by stabilizing the enzyme through dimer interactions. Thus, the mutual sharing of the active sites in EhME likely helps retain the proper conformation of the active site, contributing to the overall structural integrity and functional efficiency of the enzyme.

### 3.7. Metal-Binding Residues in EhME

Mn^2+^ or Mg^2+^ ions are essential for the catalysis of malic enzymes, not only for structural stability but also for the proper positioning of malate and oxaloacetate substrates [32]. In EhME, two key metal-binding motifs have been identified: ^165^X_h_(Q/N)X_h_EDX_h_^170^ and ^191^(W/F)(H/N)DDX^195^ (Figure 6). The metal ion is octahedrally coordinated by Glu^168^, Asp^169^, Asp^194^, two carboxylate groups from the substrates, and a water molecule (Figure 7B) [33]. Among these acidic residues, Asp^194^ plays a particularly crucial role in metal binding [34]. In the EhME structure (Figure 7B), a water molecule occupies the putative metal-binding site, bridging hydrogen bonding interactions between the protein and a glycerol molecule. Notably, Gln^195^, a residue in the second metal-binding motif, is replaced by Ile in metazoan MEs. This substitution may alter the charge density around Asp^194^, potentially affecting the metal-binding affinity. Additionally, Asp^193^ is predicted to influence the protonation state of Lys^120^, facilitating the role of Lys^120^ as the catalytic base [34].

### 3.8. Coenzyme Specificity of EhME

The coenzyme binding region in malic enzymes is highly conserved, although the preference for either NADP⁺ or NAD⁺ is determined by specific amino acid sequences [35]. Kinetic analysis of EhME reveals its preference for NADP⁺, with a K_m_ value of 0.57 ± 0.08 mM.

**Figure 8:**
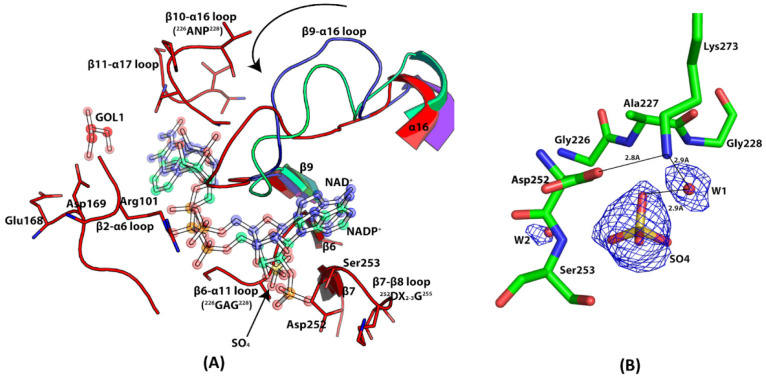
Coenzyme specificity of EhME. (**A**). The coenzyme binding region in EhME (red) is shown comparing NAD^+^ bound PhoME (Blue), and NADP^+^ bound PglME (green). β9- α16 loop undergoes close to open conformation to facilitate NADP^+^ binding. The sulfate position in EhME is shown with an arrow. DSKG and GAG motif interacts with the 2′ adenosine ribose phosphate. Conserved Arg^101^ interacts with the pyrophosphate moiety. Nicotinamide ribose interacts with the ANP motif, and the β11- α17 loop interacts with the nicotinamide moiety. A glycerol (GOL1) molecule occupies the catalytic center. Asp^168^ and Asp^169^ of the metal binding site are also shown. (**B**). The F_o_-F_c_ difference density for the sulfate ion in EhME is shown. The map is contoured at 2.9σ cut off. The sulfate molecule directly interacts with Ser^253^ and participates in water-mediated interaction with Lys^273^.

The NADP⁺ binding site was identified by comparing the EhME structure with that of NADP⁺-bound eukaryotic ME (PDB: 1GQ2) and the bacterial PhoME-NAD complex (PDB: 2DVM) (Figure 8A). This comparison suggests that the loop connecting β9 and α15 undergoes a reorientation to accommodate NADP⁺ binding in EhME. The adenosine moiety of NADP⁺ interacts with a cleft formed by the C-terminal ends of β6 and β7, primarily through hydrophobic interactions. The adenosine 2′ ribose phosphate is positioned through interactions with the ^252^DX_2_-_3_G^255^ motif in the β7-β8 loop and the ^226^GAG^228^ motif in the β6-α11 loop. This placement is further supported by the presence of a sulfate ion near these motifs (Figure 8B). The sulfate is in hydrogen bonding proximity to Ser^253^ (from the ^252^DX_2_-_3_G^255^ motif) and Ala^227^ (from the ^226^GAG^228^ motif), as well as Lys^273^. Both Ser^253^ and Lys^273^ are crucial for the specificity of NADP⁺ binding in malic enzymes [36]. The pyrophosphate group of NADP⁺ interacts with Arg^101^ from the β2-α6 loop. Additionally, the β10-α16 loop, containing the ^327^ANP^329^ sequence, is located near the nicotinamide ribose group, helping orient the nicotinamide ring properly [37]. The loop ^348^TGRXDXPNQ^356^, linking β11 and α17 in EhME, is responsible for positioning the nicotinamide moiety. In contrast, the corresponding loop in eukaryotic MEs forms a β-turn, which is absent in EhME (Figure 6). The flexibility of this region in EhME may allow the nicotinamide ring of NADP⁺ to adopt two different conformations, as observed in AsmME [38]. A recent molecular dynamics simulation study found that the cofactor can also take collapsed conformation while binding to ME, as observed in NADP(H) phosphatase [30,39].

## 4. Discussion

The malic enzyme (ME) from parasitic protozoa *E. histolytica* is suggested to be associated with the prodrug Metronidazole (Mtz). The closely related *T. vaginalis* Mtz-resistant strain has also been reported to alter its ME expression pattern, which is a secretory protein [40,41]. Upon characterization, EhME was found to be NADP⁺-specific and ATP-inhibited during oxidative decarboxylation. ATP is known to act as a competitive inhibitor in both human mitochondrial malic enzyme (HumME) and *T. vaginalis* (Tv) hydrogenosomal malic enzyme (TvME) with respect to both NADP⁺ and L-malate [42,43,44]. However, kinetic analysis of recombinant EhME (rEhME) indicates that ATP exhibits non-competitive inhibition with NADP⁺ and uncompetitive inhibition with L-malate. Docking studies support that ATP binds at the dimer interface and affects the active site via the α2-β1 loop (Figures S4 and S5). The ATP-mediated inhibition of the malic enzyme suggests a feedback regulation mechanism in the metabolic pathway [17]. Interestingly, while malic enzyme activation is not entirely negated by the deficiency of PFOR, a malic enzyme-dependent activation of Mtz has been reported in Tv [5]. A similar Mtz activation by EhME via this alternative pathway is also expected in *E. histolytica* (Eh). Complementation assays and E-tests (Figure 2A,B) demonstrate that overexpression of EhME in bacterial cells increases their susceptibility to Mtz.

Furthermore, EhME expression in Eh cells increases in a dose-dependent manner upon exposure to Mtz, as demonstrated by sqRT-PCR and Western blot analyses (Figure 2C and Figure S1). A similar trend was observed in Tv, where malic enzyme expression was markedly higher compared to other enzymes involved in Mtz activation. However, this expression declines once the cells develop resistance to Mtz [4]. From these findings, we conclude that EhME, along with enzymes like nitroreductase and PFOR, plays a crucial role in Mtz activation in Eh. The elevated expression of malic enzyme is likely a result of feed-forward regulation, continuing until the available Mtz is metabolized, albeit at the parasite’s own peril. In response to a lowering pH, EhME is secreted to the extracellular space, where it would typically reside in cytosolic vesicles (Figure 3A). Similarly to some bacterial metabolic enzymes involved in pathogenesis [45], EhME lacks a signal peptide, making its secretion process unique. A hydrogenosomal homolog of EhME, AP65 from Tv, exhibits similar properties [46]. As EhME is the only malic enzyme found in Eh, we aimed to determine if it shares functionality with its counterparts. Confocal microscopy confirmed the cell adhesion properties of rEhME. Upon incubation of rEhME with Eh cells or human enteric cells (Caco-2 and HT29), it was evident that EhME binds efficiently to both. This property is vital for Eh as it likely aids in the parasite’s ability to attach to host cells, complementing other attachment proteins such as Gal/GalNAc lectins [47].

To investigate the functional characteristics of EhME further, we determined its structure. Unlike most metazoan malic enzymes, which are typically homotetramers [36], EhME exists strictly as a dimer, with the two protomers deeply entangled. The N- and C-terminal regions of each protomer come into close proximity, forming a loop-like structure that secures a portion of the second protomer, resulting in a knot-like arrangement. This polypeptide chain entanglement is classified as type II entanglement [48]. Such unique entangled morphology has not been observed in eukaryotic malic enzyme structures reported thus far. However, a similar entangled structure has been identified in the malic enzyme from Candidatus, a prokaryotic organism [9].

While knotted folds within a single polypeptide chain have been known for over a decade [49], inter-monomer knotted structures are much rarer. Recent studies [50,51] classify EhME among a select group of proteins exhibiting this unique structural feature. Notably, most knotted proteins identified so far are enzymes [52], and these knots are far from evolutionary anomalies–instead, they often confer significant functional advantages. For instance, in ubiquitin hydrolase, a knotted structure helps the enzyme evade the proteasome, simultaneously releasing its cargo for degradation [52]. In alkaline phosphatase, a slip knot enhances thermostability [53]. Similarly, in carbonic anhydrase [54] and phytochrome [55], knotted regions contribute to greater mechanical strength and stability. In EhME, the knotted structure appears to enhance structural resilience, allowing the enzyme to withstand mechanical and environmental stresses associated with its secretion. While the precise functional role of this knot remains unclear, it is evident that it helps maintain catalytic efficiency while ensuring a compact architecture. It is plausible that EhME catalyzes the conversion of malate to pyruvate at the Host–Pathogen interface, where the resulting pyruvate (pKa ≈ 2.5) may contribute to local pH reduction. This acidification could, in turn, trigger the release of pathogenic cytolytic proteins [31].

Unlike eukaryotic malic enzymes, EhME lacks the β1-β1′ interaction at its dimeric interface. Instead, the corresponding slot between the domain A shaft and the core structure is occupied by the α5, α6, and α7 helices of the same polypeptide chain. Additionally, the N- and C-terminal regions of eukaryotic MEs are less compact compared to EhME. The NADP⁺-specific conserved motifs are located within domain C, and structural superimposing the holo-EhME with the apo-structure of pigeon liver ME confirms that the NADP⁺ binding region is conserved (Figure 8A). Notably, a sulfate molecule in the crystal structure mimics the 2′-ribose phosphate of NADP⁺ at its binding site (Figure 8B). The metal binding site of EhME is highly conserved, as demonstrated by multiple sequence alignment (Figure 6). The enzyme’s active site, located within the protein’s core, is surrounded by helices from both monomers. Key catalytic residues within the active site are conserved, and a glycerol molecule was observed bound at this site. The evolution of EhME’s unique knotted structure grants it remarkable functional versatility. However, further structure-centric functional studies are needed to fully elucidate the implications of this feature.

## Figures and Tables

**Table 1:** Data Collection and Refinement Statistics.

Parameters	Native Data	Platinum Data
Data collection and cell parameters		
Wavelength (Å)	1.5418	1.5418
Resolution Range (Å) ^1^	19.85–2.25 (2.33–2.25)	44.45–2.90 (3.00–2.90)
Space Group (Å)	P4_3_2_1_2	P4_3_2_1_2
Unit Cell Parameters (Å, °)	a = b = 117.73, c = 157.62	a = b = 117.93, c = 153.63
Total number of reflections ^1^	1,394,718 (103,047)	535,258
Unique number of reflections ^1^	53,238 (5225)	25,354
Completeness (%) ^1^	100 (100)	100 (100)
R_merge_ (%) ^1^	6.3 (24.2)	6.9 (19.0)
I/σ(I)	32.3 (12.1)	18.2 (8.4)
Redundancy	26.20 (19.49)	21.11 (20.34)
Number of monomers in asymmetric unit (Z)	2	2
Phasing		
Number of Pt sites		4
R_cullis_ (%)		88.2
R_ano_ (%)		91
Phasing power iso (acentric/centric)		0.746/0.708
Phasing power ano		0.638
FOM iso (acentric/centric)		0.176/0.207
Refinement Statistics		
Wilson B factor (Å^2^)		32.6
R_work_/R_free_		17.9/24.4
R.M.S.D. Bond lengths (Å)		0.02
R.M.S.D. Bond angle (°)		1.83
Average B factor (Å^2^)		29.0
Number of Protein atoms		7466
Number of Ligand atoms		137
Number of Solvent atoms		582
**Ramachandran Plot**		
Favored (%)		95.9
Additionally allowed (%)		3.7
Generously allowed (%)		0.0
Disallowed (%)		0.4

^1^ Values in parenthesis corresponding to highest resolution shell; $R_{merge}=\frac{\sum_{hkl}\sum_{j}\left|I_{hkl}-\langle I_{hkl}\rangle \right|}{\sum_{hkl}\sum_{j}I_{hkl,j}}$, where *I_hkl_* is the intensity of the *hkl* plane and <*I_hkl_*> is the average $R_{work}=\frac{\sum_{hkl}\left|F_{hkl}^{obs}-F_{hkl}^{calc}\right|}{\sum_{hkl}F_{hkl}^{obs}}$, where $F_{hkl}^{obs}$ is the observed structure factor, and $F_{hkl}^{calc}$ is the calculated structure factor of the *hkl* plane, and R_free_ is the 5% of the total unique reflection excluded from the refinement.

**Table 2:** Inter-monomer interacting residues of entangled polypeptide region (distance cut off 4.0 is considered).

Chain B	Chain A	Distance (Å)
B/Ala^6^/O	A/Trp^60^/NE1	2.9
B/Leu^8^/O	A/Asn^59^/ND2	3.0
B/Tyr^32^/OH	A/Asn^62^/ND2	3.6
B/Glu^34^/OE1	A/Arg^71^/NH2	3.2
B/Glu^37^/OE1	A/Arg^71^/NH1	2.8
B/Glu^37^/OE1	A/Asn^75^/ND2	2.8
B/Lys^44^/O	A/Lys^52^/NZ	2.8
B/Lys^46^/O	A/Lys^52^/N	2.9
B/Lys^46^/NZ	A/Tyr^64^/O	2.8
B/Gln^48^/NE2	A/Pro^51^/O	3.4
B/Ile^49^/N	A/Asp^131^/OD1	2.9
B/Asp^105^/OD1	A/Ser^70^/OG	2.9
B/Glu^118^/OE1	A/Leu^88^/N	3.1
B/Lys^120^/NZ	A/Tyr^65^/OH	2.8
B/Lys^125^/NZ	A/Ile^49^/O	3.3
B/Asp^131^/OD2	A/Ile^49^/O	2.9
B/Thr^161^/OG1	A/Cys^136^/N	2.7

## Data Availability

The accession number for the coordinates and structure factors for the crystal structures of *Entamoeba histolytica* malic enzyme reported in this paper is PDB: 3NV9.

## Supplementary Materials

The following supporting information can be downloaded at https://www.mdpi.com/article/10.3390/pathogens14030277/s1, Figure S1: Western blot analysis of EhME expression. Figure S2: Pairwise sequence alignment between EhME; Figure S3: The unbiased electron density map of the knot region.; Figure S4: ATP docking at the dimer interface of EhME, Figure S5: A cross-eyed stereo view of ATP docking.

## Abbreviations

The following abbreviations are used in this manuscript:

Eh	*Entamoeba histolytica*
EhME	*Entamoeba histolytica* malic enzyme
Mtz	Metronidazole
FITC	Fluorescein isothiocyanate
NADPH	Nicotinamide adenine dinucleotide phosphate (reduced)
Ni-NTA	Nickel Nitrilotriacetic acid
TRITC	Tetramethylrhodamine isothiocyanate
DAPI	4′,6-diamidino-2-phenylindole
