# Supplementary material for: Metronidazole Activation by a Deeply Entangled Dimeric Malic Enzyme in Entamoeba histolytica
Source: Pathogens. 2025 Mar 13;14(3):277. doi: 10.3390/pathogens14030277 (PMC11944484; doi:10.3390/pathogens14030277)
Supplement: Supplementary file 1 [file pathogens-14-00277-s001.zip › pathogens-3487579-supplementary.pdf]

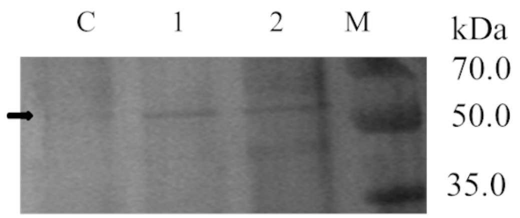

Figure S1: Western blot analysis of EhME expression. Western blot was done from total protein isolated form untreated (C), Metronidazole treated 5μM (5) and 12μM (12) cells. Lane M indicates molecular weight marker. Arrow indicating the expressed protein position.

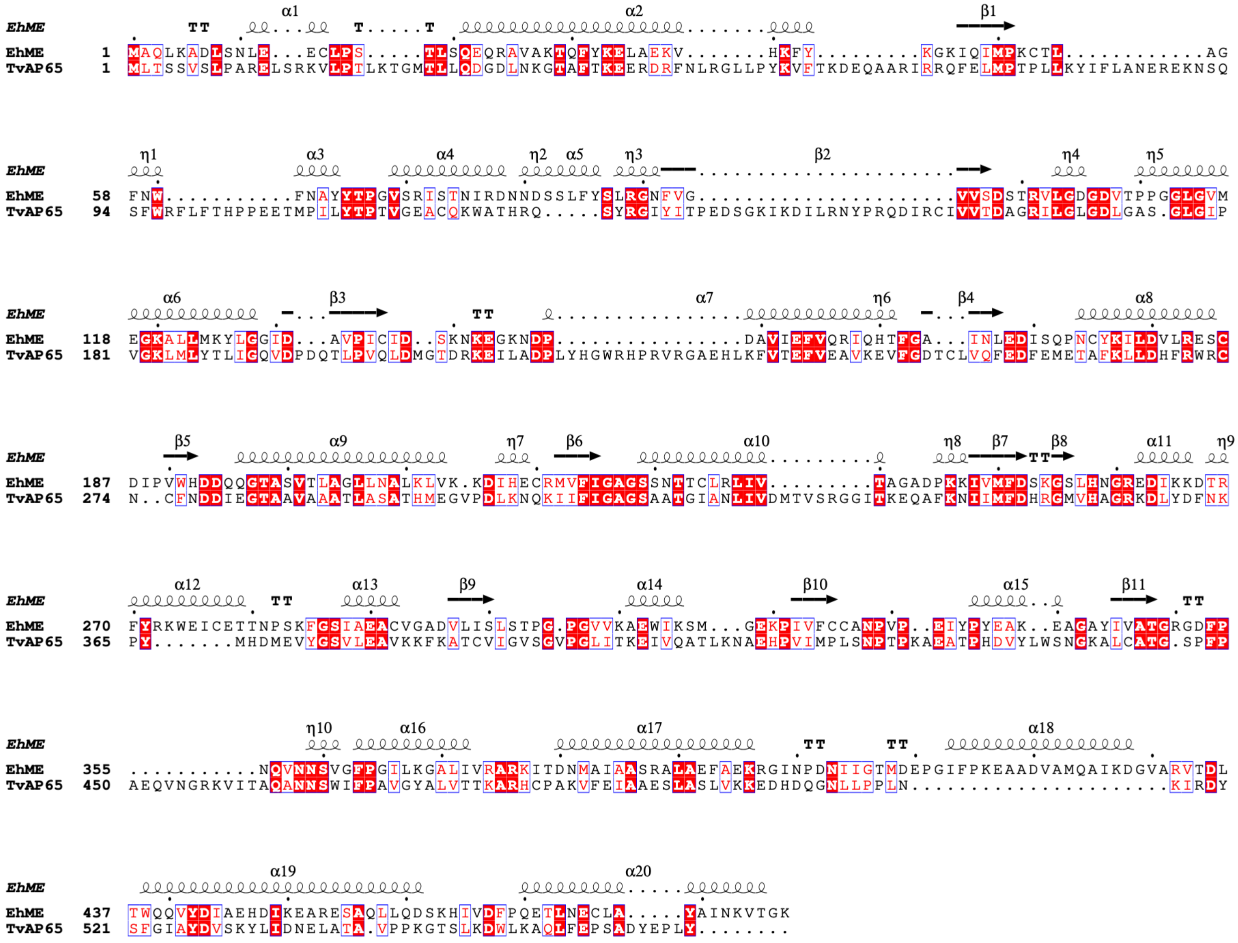

Figure S2: Pairwise sequence alignment between EhME (Q9NH04) and TvAP65-1 (A2EKE3) sharing 30% identity.

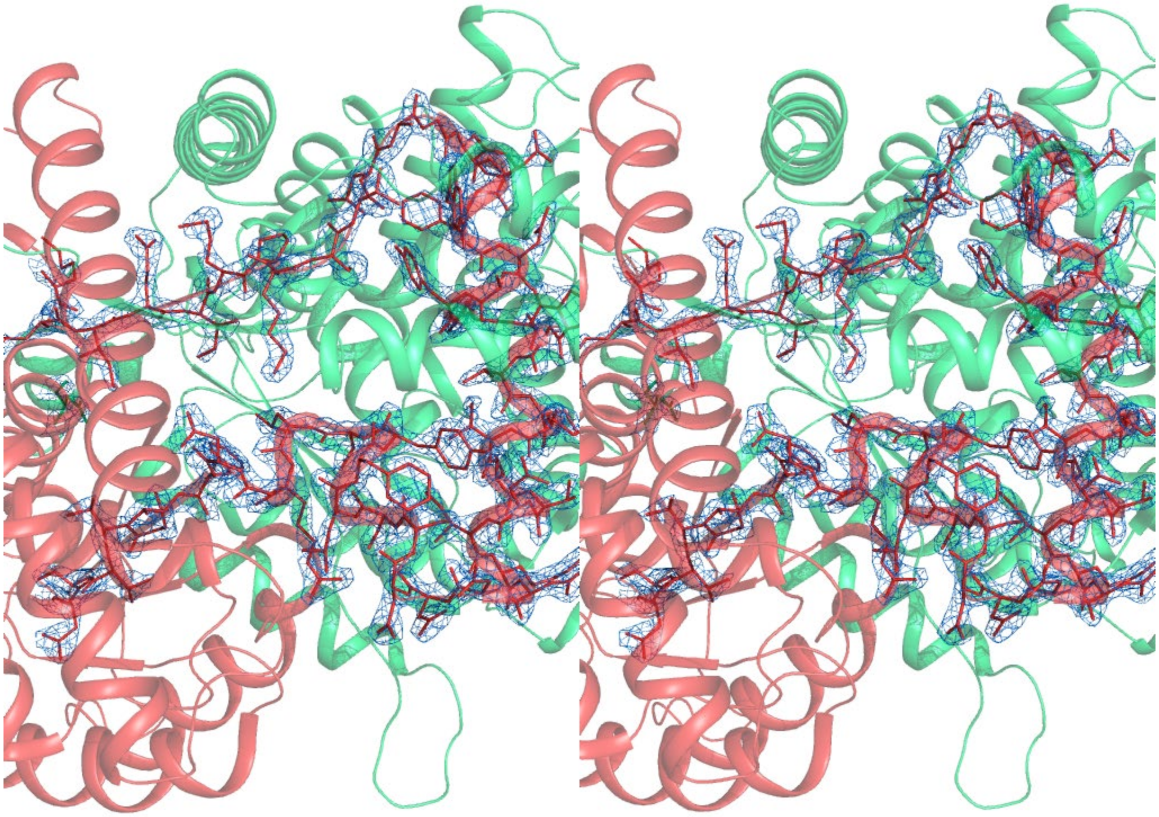

Figure S3: The unbiased electron density map of the knot region. The Fo-Fc map is contoured at 2.5 $\boldsymbol{\sigma}$. Colors represent different chains.

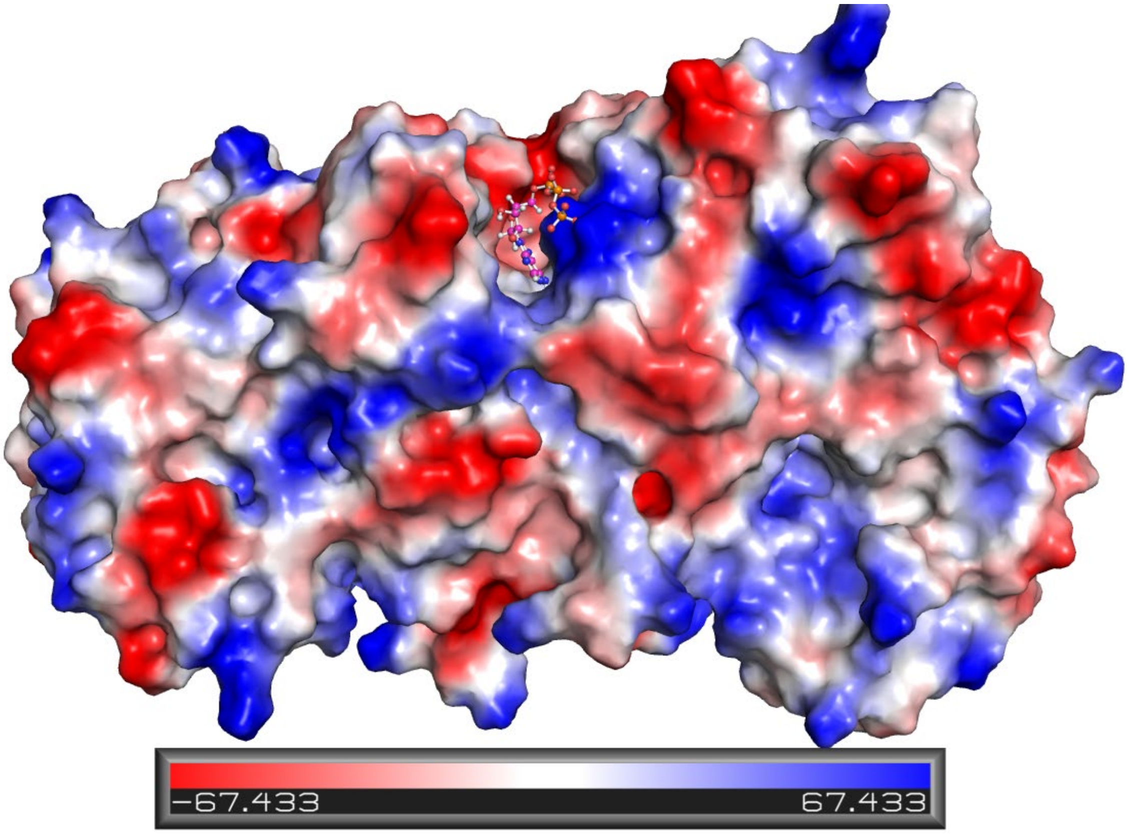

A

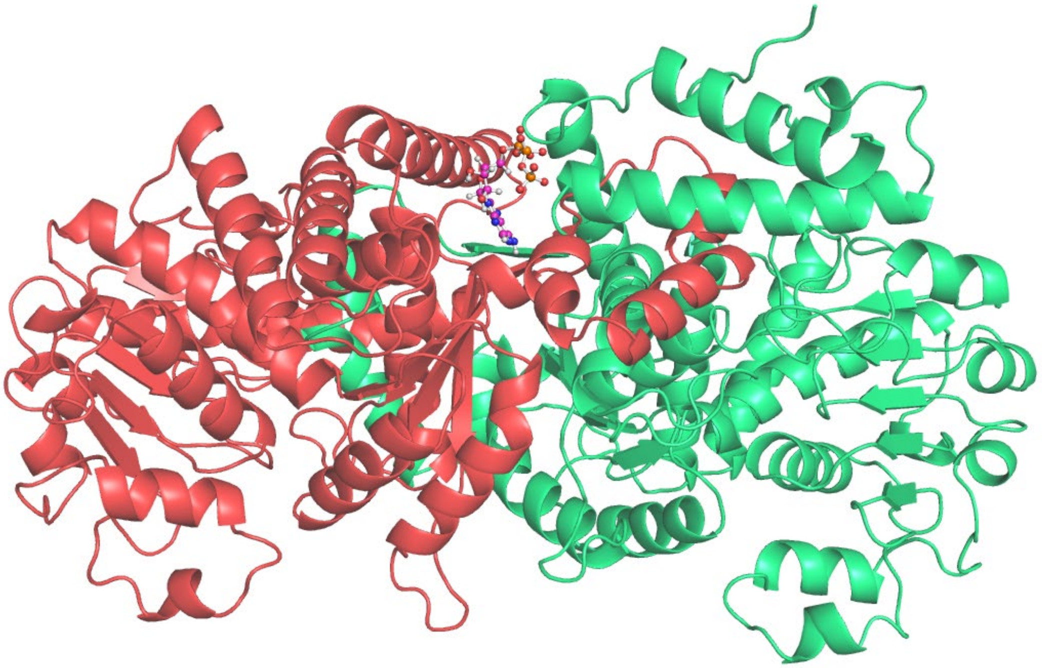

B

Figure S4: ATP docking at the dimer interface of EhME. A) the surface charge representation, B) Cartoon representation.

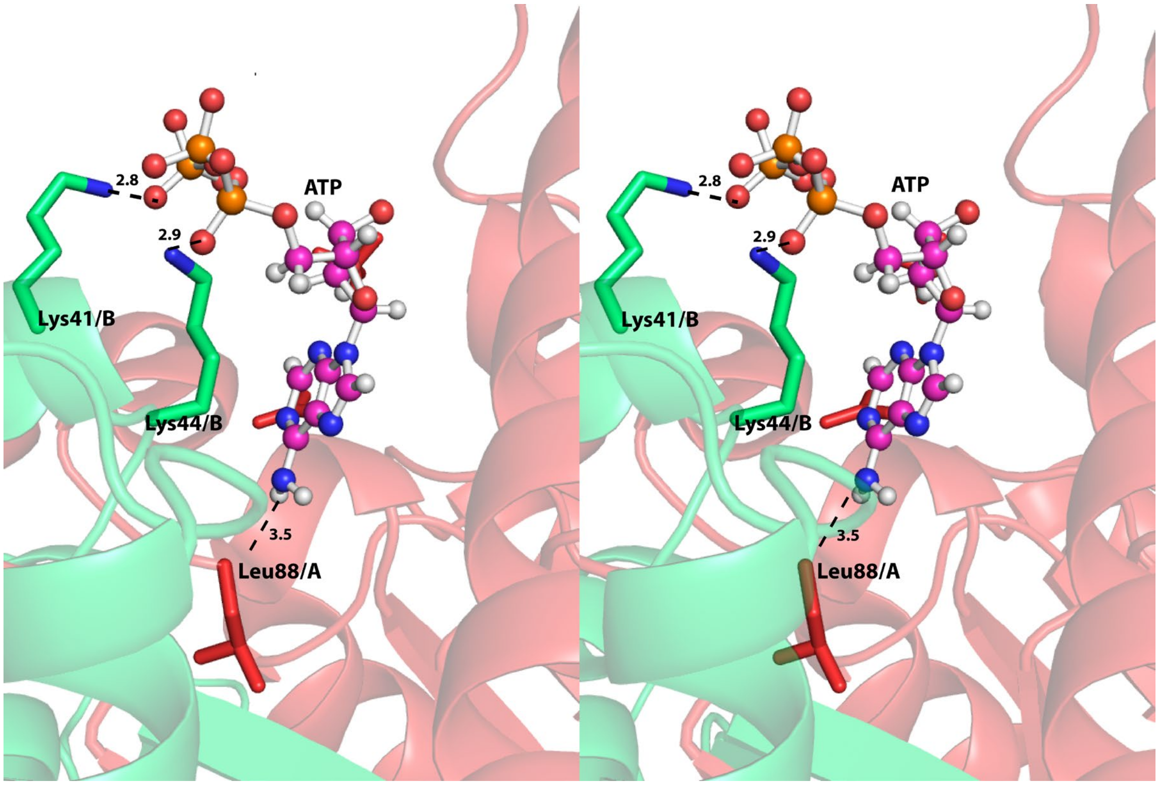

Figure S5: A cross-eyed stereo view of ATP docking at the dimer interface of EhME. Lys residues are the part of α2-β1 loop. Adenosine –amine is also within interacting distance of Leu88, which is a part of α5'.
